# Transoral laser microsurgery for supraglottic carcinomas: results of a prospective multicenter trial (SUPRATOL)

**DOI:** 10.3389/fonc.2024.1440024

**Published:** 2024-09-20

**Authors:** Petra Ambrosch, Sylvia Meuret, Andreas Dietz, Asita Fazel, Rainer Fietkau, Ralf Tostmann, Ursula Schroeder, Anne Lammert, Julian Künzel, Martin C. Jäckel, Daniel Boeger, Claudia Scherl, Thomas Deitmer, Kerstin Breitenstein, K.-Wolfgang Delank, Hermann Hilber, Sarah Vester, Stephan Knipping, Ulrich Harreus, Matthias Scheich, Sylva Bartel, Stefan K. Plontke, Sven Koscielny, Johannes A. Veit, Jens Greve, Volker Schilling, Maximilian Linxweiler, Sonja Weiß, Georgios Psychogios, Christoph Arens, Claus Wittekindt, Jens Oeken, Maria Grosheva, Christoph Borzikowsky

**Affiliations:** ^1^ Department of Otorhinolaryngology, Head and Neck Surgery, University Hospital Schleswig-Holstein (UKSH), University of Kiel, Kiel, Germany; ^2^ Section of Phoniatrics and Audiology, Clinic of Otorhinolaryngology, University of Leipzig, Leipzig, Germany; ^3^ Clinic of Otorhinolaryngology, University of Leipzig, Leipzig, Germany; ^4^ Department of Radiooncology, Friedrich-Alexander-University, Erlangen-Nuremberg, Erlangen, Germany; ^5^ Clinical Trial Unit UMG, Universitätsmedizin Goettingen, Goettingen, Germany; ^6^ Department of Otorhinolaryngology, Head and Neck Surgery, University Hospital Schleswig-Holstein (UKSH), University of Luebeck, Luebeck, Germany; ^7^ Department of Otorhinolaryngology, Head and Neck Surgery, Medical Faculty Mannheim, University Hospital Mannheim, University of Heidelberg, Mannheim, Germany; ^8^ Department of Otorhinolaryngology, Head and Neck Surgery, University Hospital of Regensburg, Regensburg, Germany; ^9^ Department of Otorhinolaryngology, Helios-Kliniken Schwerin, Schwerin, Germany; ^10^ Department of Otorhinolaryngology, SRH Zentralklinikum Suhl, Suhl, Germany; ^11^ German Society of Otorhinolaryngology, Head and Neck Surgery (DGHNO-KHC), Bonn, Germany; ^12^ Department of Otorhinolaryngology, Helios-Klinikum Erfurt, Erfurt, Germany; ^13^ Department of Otorhinolaryngology, Head and Neck Surgery, Klinikum der Stadt Ludwigshafen gGmbH, Ludwigshafen, Germany; ^14^ Department of Otorhinolaryngology, Head and Neck Surgery, University Hospital of Regensburg and Private Medical Practice for Otorhinolaryngology, Regensburg, Germany; ^15^ Department of Otorhinolaryngology, Head and Neck Surgery, Städtisches Klinikum Dessau, Dessau, Germany; ^16^ Department of Otorhinolaryngology, Krankenhaus Bad Tölz, Bad Tölz, Germany; ^17^ Department of Otorhinolaryngology, Plastic, Aesthetic and Reconstructive Head and Neck Surgery, Julius-Maximilians-University Hospital Würzburg, Würzburg, Germany; ^18^ Department of Otorhinolaryngology, Head and Neck Surgery, Martin Luther University Halle-Wittenberg, Halle, Germany; ^19^ Department of Otorhinolaryngology, Head and Neck Surgery, University Hospital Jena, Jena, Germany; ^20^ Private Medical Practice for Nasal Surgery, Muenchen, Germany; ^21^ Department of Otorhinolaryngology, Head and Neck Surgery, Ulm University Hospital, Ulm, Germany; ^22^ Department of Otorhinolaryngology, Head and Neck Surgery, Vivantes Klinikum Neukölln, Germany; ^23^ Department of Otorhinolaryngology, Saarland University, Homburg, Germany; ^24^ Department of Otorhinolaryngology, Klinikum Kassel, Kassel, Germany; ^25^ Clinic of Otorhinolaryngology, University of Ioannina, Ioannina, Greece; ^26^ Department of Otorhinolaryngology, Head and Neck Surgery, University of Giessen, Giessen, Germany; ^27^ Department of Otorhinolaryngology, Head and Neck Surgery, Klinikum Dortmund, Dortmund, Germany; ^28^ Department of Otorhinolaryngology, Hospital Chemnitz, Chemnitz, Germany; ^29^ Department of Otorhinolaryngology, Head and Neck Surgery, University of Cologne, Cologne, Germany; ^30^ Institute of Medical Informatics and Statistics, Christian-Albrechts-University Kiel, Kiel, Germany

**Keywords:** transoral laser microsurgery, supraglottic carcinoma, functional outcomes, FEES, MDADI, VHI, prospective multicenter trial

## Abstract

**Background:**

A limited number of single institutions have published retrospective cohort studies on transoral laser microsurgery for supraglottic laryngectomy (TLM-SGL). These studies have shown that the oncologic outcomes of TLM-SGL are comparable to those of open SGL. However, there is limited information available regarding swallowing rehabilitation and quality of life (QoL).

**Patients and methods:**

SUPRATOL is a prospective, multicenter trial assessing the functional outcomes of TLM-SGL +/− adjuvant radio-(chemo)-therapy. The primary endpoint was aspiration-free swallowing at 12 months, as established using fibreoptic endoscopic evaluation of swallowing (FEES) and defined as a grade < 6 on the penetration–aspiration scale. Secondary endpoints were swallowing- and voice-related QoL, the prevalence of temporary and permanent tracheostomy and percutaneous gastrostomy, local control, laryngectomy-free survival, overall survival, and disease-free survival, as well as the influence of treatment centers on outcomes.

**Results:**

From April 2015 to February 2018, 102 patients were recruited from 26 German Otorhinolaryngology (ORL) hospitals. All patients had TLM-SGL and 96.1% underwent uni- or bilateral, mostly selective neck dissection. To 47.0% of patients, adjuvant radio-(chemo)-therapy (R(C)T) was administered. The median follow-up period was 24.1 months. At 12-month follow-up, completed by 84.3% of patients, 98.2%, 95.5%, and 98.8% were free of aspiration when tested with saliva, liquid, or pulp. Adjuvant R(C)T, pT category, and type of resection had no significant influence on swallowing rehabilitation. A total of 40.2% of patients had been tracheotomized, and in 46.1% of patients, a PEG tube was inserted. At the 24-month follow-up, 5.3% of patients still required a tracheostomy, and 8.0% continued to use a percutaneous endoscopic gastrostomy (PEG) tube. Deterioration of swallowing- and voice-related QoL was observed immediately after treatment, but patients recovered, and baseline values were reached again. The Kaplan–Meier 2-year rates for local control, laryngectomy-free survival, overall survival, and disease-free survival were 88%, 92%, 93%, and 82%, respectively.

**Conclusions:**

Our prospective multicenter trial shows that, at 12 months post-TLM-SGL +/− R(C)T, 95.5%–98.8% of patients achieved aspiration-free swallowing. Morbidity was higher than previously reported. The rates of permanent tracheostomy and gastrostomy tube placement correspond to previous cohort studies. The 2-year oncologic outcomes are within the reported range.

**Clinical trial registration:**

https://drks.de/search/en/trial/DRKS00004641, identifier (DRKS00004641).

## Introduction

The goal of developing transoral laser microsurgery for supraglottic laryngectomy (TLM-SGL) has been to reduce surgical morbidity and improve organ preservation and function while maintaining oncologic outcomes. TLM is now well established in many German ORL hospitals and is incorporated into the German S3 guideline on the diagnosis, treatment, and follow-up of laryngeal cancer (1). A limited number of mainly European specialized single institutions have published retrospective cohort studies (2–11). It has been shown that the oncologic outcomes of TLM-SGL are comparable to those of open SGL, and that TLM is associated with acceptable surgical morbidity. However, these retrospective studies provide very limited information on functional aspects, such as swallowing rehabilitation and quality of life issues. A recent systematic review revealed a paucity of objective measures and considerable data heterogeneity, which limited the comparison of functional outcomes between partial supraglottic resection and radiotherapy (12).

TLM has never been tested in multicenter trials or directly compared to the conventional surgical treatment standard. It is difficult to substantiate this in a randomized trial for various reasons: recruiting patients for historical comparison with open partial laryngectomy is ethically unacceptable because of the well-known greater morbidity of open surgery. The comparison of TLM with transoral robotic surgery (TORS) did not seem possible because TORS had hardly been evaluated for supraglottic cancer at the time of the inception of the trial protocol and was not established in a relevant number of German ORL hospitals. Finally, supraglottic laryngeal carcinoma is a rare tumor, which makes multiarm studies difficult to realize.

To date, there have been no prospective clinical trials investigating functional outcomes. We therefore performed a prospective, nonrandomized, multicenter trial to clarify whether the positive results of TLM-SGL reported in monocentric retrospective studies can be confirmed in a multicenter setting. The focus of interest was set on functional aspects, such as postoperative swallowing rehabilitation (primary endpoint). Further aims were the evaluation of oncological parameters, quality of life (QoL), morbidity of treatment, and the influence of treatment center on outcomes (secondary endpoints).

## Patients and methods

SUPRATOL is an investigator-initiated, nonrandomized, single-arm, multicenter trial designed to evaluate the functional outcomes of TLM-SGL. The trial protocol (13) was approved by the ethics committee of the Medical Faculty of Kiel University (A149/14) and the ethics committees of the participating institutions. The trial was registered with the German Clinical Trial Register (DRKS00004641). Participation in the study was open to all German ORL hospitals meeting the certification criteria for head and neck cancer centers as defined by the German Cancer Society.

### Patients

Eligible patients were at least 18 years of age and had a histologically confirmed diagnosis of squamous cell carcinoma (SCC) of the supraglottic larynx, staged T2-3 N0-3 M0 (UICC 7th ed., 2010) (14). Exclusion criteria included previous treatment for head and neck SCC, previous malignant disease, inability to achieve R0 resection of neck metastases, simultaneous distant metastases, simultaneous second primary tumor (SPT), and tumor-unrelated swallowing dysfunction. All patients provided written informed consent.

### Endpoints

The primary endpoint was aspiration-free swallowing at 12 months, as established using fibreoptic endoscopic evaluation of swallowing (FEES) and defined as a grade < 6 on the penetration–aspiration scale (PAS) according to Rosenbek (15). Secondary endpoints were local control, laryngectomy-free survival, overall survival, and disease-free survival, swallowing- and voice-related quality of life, complications of treatment, severe adverse events (SAEs), the prevalence of tracheostoma and of PEG-tube feeding, and the influence of treatment center on outcomes.

### Assessments and outcomes

Before inclusion in the study, staging was performed through clinical examination, panendoscopy, and biopsy, as well as CT or MRI scans of the primary site and neck, CT scans of the chest, and ultrasound imaging of the abdominal organs. Preoperatively, at baseline, standardized FEES was performed. The swallowing-related QoL was assessed using the MD Anderson Dysphagia Index (MDADI) (16), and the voice-related QoL was evaluated using the Voice Handicap Index (VHI) (17). Patients completed the MDADI and VHI questionnaires at baseline and during each follow-up visit. The MDADI composite score and the VHI total score were used for analysis. All patients were reviewed at multidisciplinary tumor boards.

### FEES

FEES were video-documented and evaluated centrally by an experienced reviewer in the Section of Phoniatrics and Audiology of the Clinic of ORL of Leipzig University. The findings were classified on the eight-point PAS (15). PAS 1 is defined as “material does not enter the airway”, PAS 2–5 as “penetration (the material does not pass below the vocal folds)”, and PAS ≥ 6 as “aspiration (the material passes below the vocal folds)”. Swallowing saliva and swallowing boluses of thin liquid and pulpy consistency, both colored with green food coloring, were tested. The reviewer selected the worst PAS for each bolus type for analysis. PAS was dichotomized for swallowing evaluation. PAS < 6 was considered “aspiration-free swallowing” and PAS ≥ 6 “aspiration”. To examine the dynamics of swallowing rehabilitation over the course of the trial at the individual level, scores were referenced to the baseline PAS at the preoperative examination. Details are provided in the **Supplementary Appendix**.

### Surgery

Surgery consisted of TLM-SGL, as described previously (2, 3), and uni- or bilateral, preferably selective neck dissection (ND). Whether a tracheostomy should be performed was left to the surgeons’ judgment. Resection margins were defined as follows: Rx, the presence of residual tumor cannot be determined; R0, margins ≥ 5 mm; R0 with “close margins” (1–5 mm); R1, microscopic positive margins; and R2, macroscopic positive margins. The final resection status, including any necessary re-resection, was documented.

### Adjuvant treatment

#### Primary site

R0 resection was not regarded as an indication for adjuvant radiotherapy (RT). In the case of R0 resection with close resection margins and a pN0 neck, the indication for RT of the laryngeal remnant was optional. Microscopic positive resection margins, despite re-resection (R1 resection), were regarded as an indication for adjuvant radiochemotherapy (RCT).

#### Neck

Multiple lymph node metastases (pN2) and lymph node metastases with extranodal spread (ENS) were regarded as indications for adjuvant RCT. In cases where there is only one lymph node metastasis without ENS (pN1, ENS^−^) and R0 resection of the primary tumor, adjuvant RT should be limited to the affected side of the neck. In the presence of a node-positive neck, the laryngeal remnant should always be irradiated. Postoperative RT was preferably IMRT with an integrated boost. The contouring of lymph node levels and organs at risk was carried out according to the consensus guidelines proposed by an international group of head and neck radiation oncologists (18). The standard chemotherapy regimen for RCT was cisplatin (200 mg/m^2^). With regard to the radiation doses to be applied, the study protocol (13) specified the following: laryngeal remnant (CTV1) pT2R0, 56 Gy; pT2 “close margins” and pT3, 60 Gy; lymph node levels with tumor involvement (CTV2) without ENS, 56 Gy and with ENS, 64 Gy; and lymph node levels without tumor involvement (CTV3), 50 Gy.

### Follow-up

**Figure 1:**
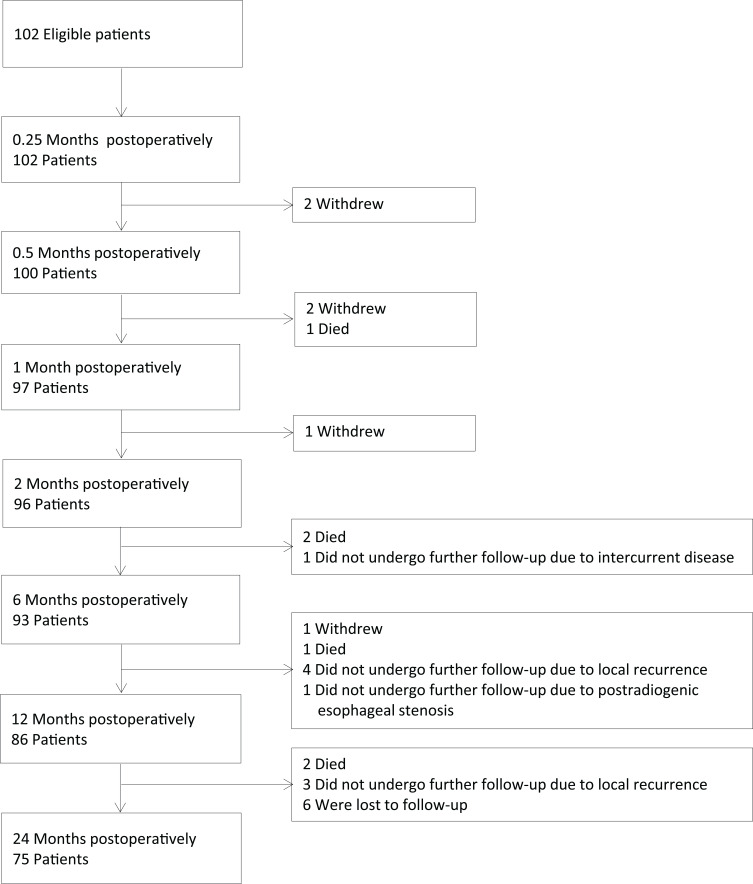
CONSORT flow diagram.

Follow-up consisted of a clinical examination for 24 months after surgery. Swallowing was assessed by FEES at baseline before surgery and at 0.25, 0.5, 1, 2, 6, 12, and 24 months after surgery. Suspected recurrences were assessed by means of biopsy/needle aspiration and pathological examination or by means of cross-sectional imaging, depending on the site of recurrence. Complications of surgery were recorded for a period of 30 days after the operation. Side effects of adjuvant RT/RCT were recorded before, in the middle, at the end, and 6 months after completion of RT/RCT using the common terminology criteria for adverse events (19). Serious adverse events were recorded up to the end of follow-up time. The CONSORT flow diagram is shown in **Figure 1**.

### Statistical methods

The trial was planned to recruit 200 patients, as this seemed possible in a recruitment period of 2 years involving 25 treatment centers (13). For all analyses, the intention-to-treat principle was used, which considers all patients in the analyses, whether they drop out or not. Demographic and clinical characteristics were recorded descriptively. Categorical variables are reported with absolute and relative frequencies. Metric variables are presented with mean values and standard deviations. Local control, laryngectomy-free survival (survival with preserved larynx), overall survival, and disease-free survival were estimated according to the Kaplan–Meier method and presented with percentages and 95% confidence intervals. The recommended scoring methods for the QoL questionnaires were applied. For categorical variables, Fisher’s exact test was used to compare categorical variables between subgroups. For metric variables, mean comparison between subgroups was done with a two-sample *t*-test for independent samples. In cases where the assumptions of this parametric test were not fulfilled, the nonparametric Mann–Whitney *U* test was used. *p*-values < 0.05 were regarded as statistically significant. For the comparison of treatment centers, the binomial test (test for equal distribution, test proportion = 0.50) and Fisher’s exact test were used to calculate the *p*-values.

## Results

### Patient characteristics and treatment

A total of 102 (range, 1–12; mean, 3.92; standard deviation [SD], 2.69) patients were recruited from 26 ORL hospitals. Local principal investigators and participating hospitals are listed in **Supplementary Table S1** in the **Supplementary Appendix**. Baseline patient characteristics are shown in **Table 1**. Detailed information is provided in **Supplementary Tables S2** and **S3** in the **Supplementary Appendix**.

**Table 1:** Patient characteristics (*n* = 102 patients).

Characteristic	
** Age** (years; mean (SD), min, max)	61.9 (8.21), 42.0, 82.0
** Male sex** (*n*; %)	70 (68.6)
Smoking history	
** **Never smoker (*n*; %)	5 (4.9)
** **Active smoker (*n*; %)	71 (69.6)
** **Former smoker (*n*; %)	26 (25.5)
Alcohol history	
** **Abstinent (*n*; %)	32 (31.4)
** **Active alcohol consumption (*n*; %)	69 (67.6)
** **Unknown (*n*; %)	1 (1.0)
cT category (*n*; %)	
** **T1	3 (2.9)
** **T2	76 (74.5)
** **T3	23 (22.6)
cN category (*n*; %)	
** **N0	54 (52.9)
** **N1	15 (14.7)
** **N2	31 (30.4)
** **N3	2 (2.0)
Clinical UICC stage (*n*; %)	
** **I	1 (1.0)
** **II	45 (44.1)
** **III	23 (22.6)
IVa	33 (32.3)

### TLM-SGL

**Figure 2:**
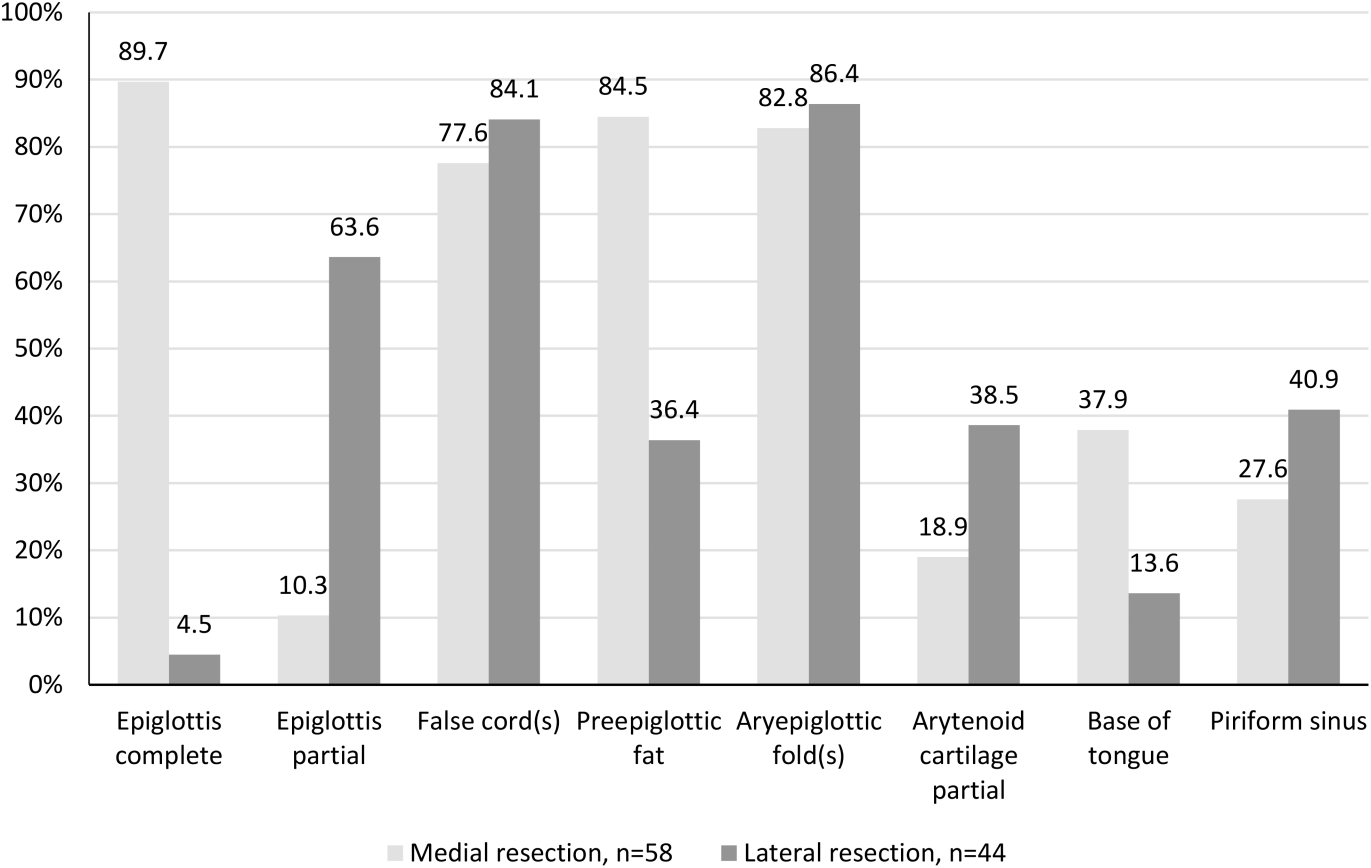
Type of transoral laser microsurgical supraglottic laryngectomy (TLM-SGL). Resected laryngeal subsites and adjacent sites in percentage (*n* = 102 patients).

With TLM, the extent of the laryngeal resection is adapted to the tumor growth. Resection types were not defined in the study protocol (13). The complete or partial resection of laryngeal structures and the extension of the resection to the base of the tongue, piriform sinus, and soft tissues of the neck were documented in tabular form. From the extent of resections performed, two groups can be derived. The “medial resection” group with bilateral removal of supraglottic structures comprised 58 (56.9%) and the “lateral resection” group with predominantly unilateral removal of supraglottic structures 44 (43.1%) cases (**Figure 2**). In 35 (34.3%) patients, elective tracheostomy was performed; in 34 of these, it was done simultaneously with TLM-SGL, and in one patient, 10 days preoperatively.

Re-resection was performed in 15 (14.7%) patients. In the final assessment, i.e., including the result of the re-resection, the supraglottic carcinoma was microscopically completely resected (R0 resection) in 94 (92.2%) patients. Microscopically positive resection margins (R1 resection) were described in three (2.9%) and nonevaluable resection margins (Rx resection) in five (4.9%) cases. Detailed information on the results of the histopathologic examination of laryngeal and ND specimens and pathologic staging is provided in **Supplementary Table S4** in the **Supplementary Appendix**.

### Neck dissection

Contrary to the requirements of the study protocol (13), elective ND was not carried out in four (3.9%) patients with clinically node-negative (N0) neck. In 98 patients, elective or therapeutic, uni- (*n* = 27, 26.5%) or bilateral (*n* = 71, 69.6%) ND was performed. In 85 (83.3%) patients, ND was performed together with the TLM-SGL. In 13 (12.8%) patients, a staged procedure was carried out. Fifty-three (52.0%) patients were histopathological node-negative (pN0) and 45 (44.1%) node-positive (pN+). In 18 of 45 (40.0%) pN+ patients, one or more metastases with ENS were diagnosed pathologically.

### Comparison of clinical and pathologic T and N staging

Despite extensive diagnostics including CT and/or MRI scans of larynx and neck in all patients, the cT and pT categories matched in only 76 (74.5%) of 102 cases. In 11 (10.8%) cases, upstaging (cT1 to pT2, 1; cT2 to pT3, 10), and in 15 (14.7%) cases, downstaging (cT2 to pT1, 9; cT3 to pT2, 6) were required. The comparison of cN and pN staging shows that 12 (24.0%) of 50 patients with cN0 neck had occult lymph node metastases, and 15 (31.3%) of 48 patients who had been clinically suspected to have metastases were free of lymph node metastases postoperatively. The results of pathologic staging are shown in **Table 2** and in detailed version in **Supplementary Table S4** in the **Supplementary Appendix**.

**Table 2:** Pathologic staging (*n* = 102 patients).

Parameter	
pT category (*n*; %)	
pT1	11 (10.8)
pT2	64 (62.7)
pT3	27 (26.5)
N/pN category (*n*; %)	
N0	4 (3.9)
pN0	53 (52.0)
pN1	12 (11.8)
pN2	33 (32.3)
Pathologic UICC stage (*n*; %)	
I	7 (6.9)
II	34 (33.3)
III	28 (27.5)
IVa	33 (32.3)

### Adjuvant treatment

After surgery, five (4.9%) of 102 patients had dropped out of the study. No adjuvant treatment was given to 49 (48.0%) patients. Of the patients who did not undergo adjuvant treatment, 39 of 49 (79.6%) had no indication for R(C)T according to the trial protocol. Indicated adjuvant treatment was not performed in five (4.9%) patients and refused by another five (4.9%) patients. Forty-eight (47.1%) patients received adjuvant RT (*n* = 24, 23.5%) or RCT (*n* = 24, 23.5%). In 47 (97.9%) of 48 patients, the laryngeal remnant was irradiated. The radiation dose to the larynx (CTV1), recommended in the trial protocol (13) in the respective treatment situation, was received by 21 (44.7%) patients; higher doses (> 60 Gy) were applied in 17 (36.2%); and lower doses (< 60 Gy, pT2R0, close resection margins) in nine (19.1%) patients. The radiation dose to the larynx was in all cases within the range usually applied in the adjuvant setting. Forty-four (91.6%) patients received uni- (*n* = 9, 18.8%) or bilateral (*n* = 35, 81.2%) radiotherapy to the neck. The total doses applied to the regional lymphatic drainage (CTV2, CTV3) corresponded to the recommendations of the trial protocol.

Concomitant chemotherapy was received in 24 (50.0%) of 48 irradiated patients. The indications were lymph node metastases with ENS in 12 (50%), multiple lymph node metastases in six (12.5%), and R1 or Rx resection in six (12.5%) patients. Cisplatin was administered in 18 (75.0%) of 24 patients. In 10 (41.6%) patients, the scheduled total dose of 200 mg/m^2^ was reached. Comorbid diseases forced dose reduction in eight (33.3%) patients. Deviations from the proposed chemotherapy standard were noted in six (25.0%) patients.

### Complications of surgical treatment

SAEs were not reported. A total of eight (7.9%) of 102 patients experienced surgical complications after TLM-SGL. Seven (6.9%) patients had a postoperative endolaryngeal hemorrhage. Endoscopic coagulation was performed in all patients, and in two (2.0%) patients, ligation of feeding vessels in the neck was done additionally. Four (3.9%) patients with postoperative bleeding and one (1.0%) patient with postoperative laryngeal edema required tracheostomies. A total of four (2.4%) of 169 NDs were complicated; two (1.2%) by postoperative hemorrhage and another two (1.2%) by deep neck infection. Revision surgery was performed in all cases, in one case with a tracheostomy.

### Side effects and complications of adjuvant treatment

Side effects of adjuvant R(C)T were documented exclusively for the parameters “mucositis”, “dysphagia”, and “voice changes”. Up to the end of R(C)T, side effects of grades 1–3 were reported. Six months postradiotherapy, postradiogenic stricture of the cervical esophagus (grade 4 adverse event) was documented in two (4.2%) of 48 patients. The adjuvant RT and RCT groups did not differ with regard to mucositis and voice changes (all *p*-values > 0.05). With regard to dysphagia, the RCT group experienced significantly more side effects than the RT-alone group at the end of therapy (*p* < 0.001) and 6 months posttherapy (*p* = 0.036). Since no other significant differences were observed, the RT and RCT groups were combined for further analyses.

### Swallowing function assessed by FEES

**Figure 3:**
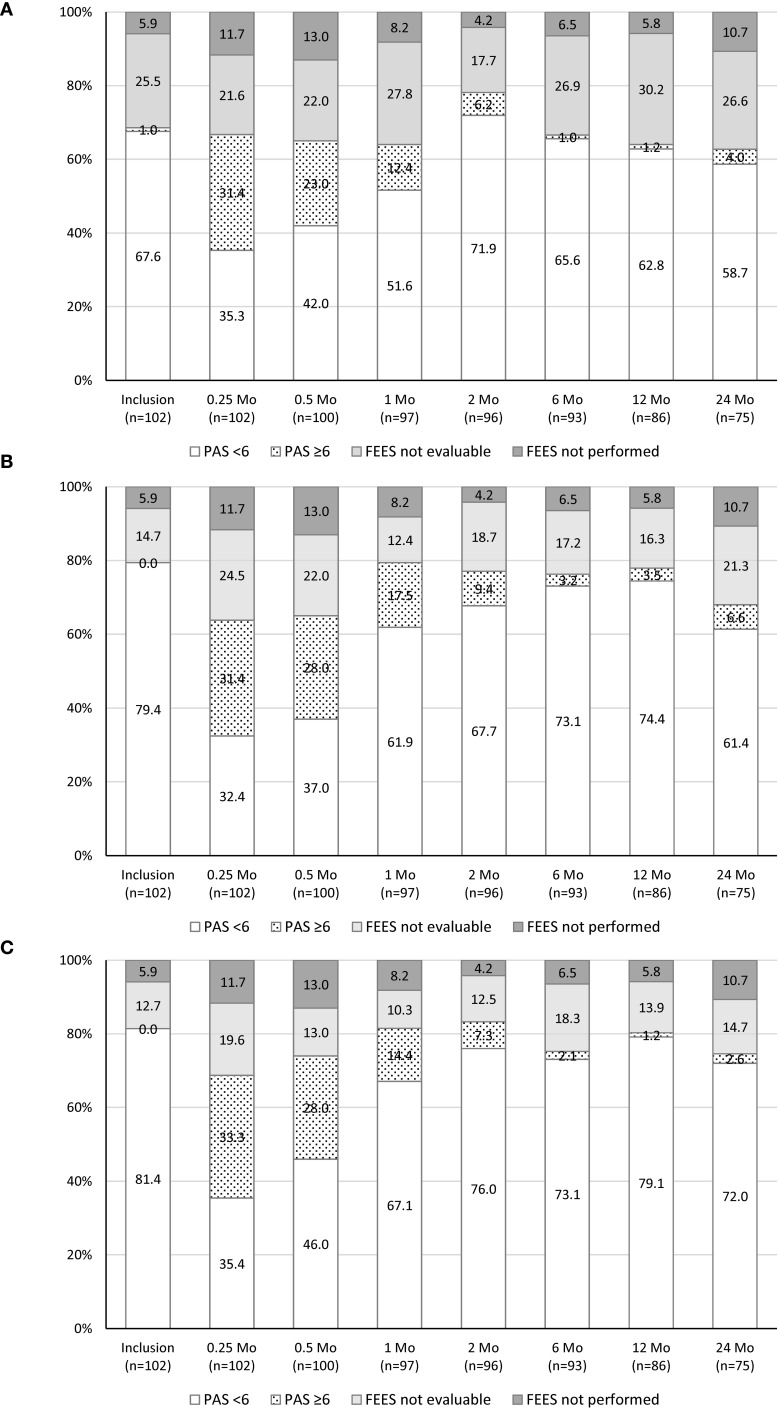
Longitudinal analysis of the distribution of dichotomized PAS in percentage (*n* = 102 patients). PAS, penetration–aspiration scale; FEES, fibreoptic endoscopic evaluation of swallowing. **(A)** Tested: saliva, PAS < 6, aspiration-free swallowing, PAS ≥ 6, aspiration. **(B)** Tested: liquid, PAS < 6, aspiration-free swallowing, PAS ≥ 6, aspiration. **(C)** Tested: pulp, PAS < 6, aspiration-free swallowing, PAS ≥6, aspiration.

Each FEES examination consisted of three parts: spontaneous, unintended swallow of saliva, and intended swallow of boluses of thin liquid and pulp. The total number of FEES examinations possible was determined by the number of patients who had completed the respective follow-up visits and amounted to 751. Of those, 438 (58.3%) examinations were fully evaluable (for three parts) and 180 (23.9%) were partially evaluable (for < 3 parts). Seventy-one (9.5%) examinations were not evaluable and 62 (8.3%) were not performed. When tested with saliva and boluses of both consistencies, aspiration was present in one-third of patients at 0.25 months postoperatively. However, swallowing function improved steadily over time. Twelve-month follow-up was completed by 84.3% (86/102) of patients. FEES with saliva was not done or was not evaluable in 36.0% (31/86) of patients, FEES with liquid in 22.1% (19/86) of patients, and FEES with pulp in 19.8% (17/86) of patients. When tested with saliva, liquid, or pulp, 98.2% (54/55), 95.5% (64/67), and 98.8% (68/69) of patients were free of aspiration. The longitudinal evaluation of the distribution of dichotomized PAS is shown in **Figures 3A–C**. To examine the influence of adjuvant treatment (no irradiation vs. R(C)T), of pT category (pT2 vs. pT3 tumor), and of type of resection (medial vs. lateral) on swallowing, the mean dichotomized PAS for saliva, liquid, and pulp were compared. Comparisons at 6- and 12-month follow-up showed no statistically significant differences for all time points and bolus consistencies (all *p*-values > 0.05). Details on the dynamics of swallowing rehabilitation on an individual basis are provided in the **Supplementary Appendix** and in **Supplementary Figures S1A–C**.

### Prevalence of feeding tube, PEG tube, and tracheostoma

**Figure 4:**
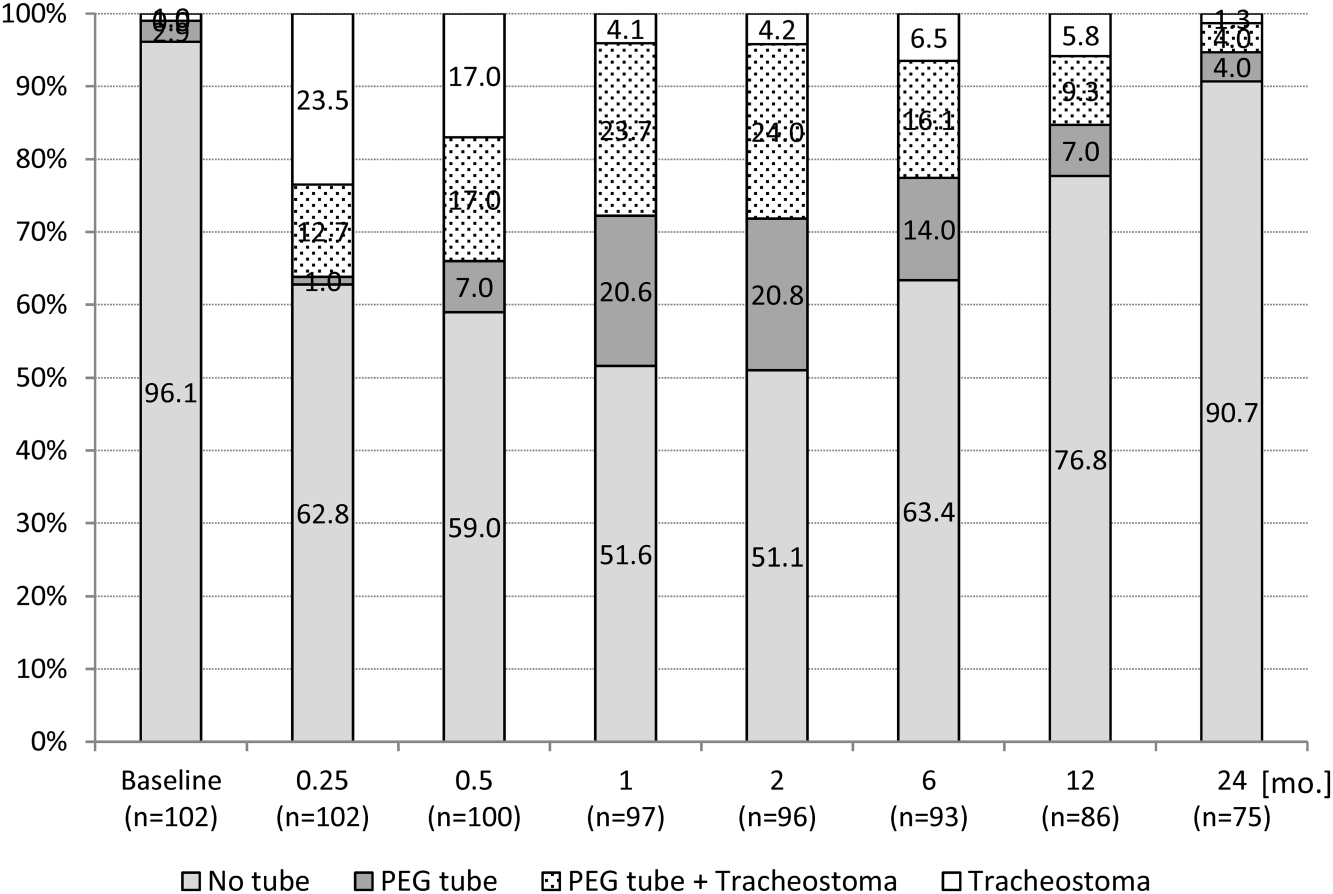
Longitudinal analysis of the prevalence of tracheostoma and PEG tube in percentage (*n* = 102 patients). PEG, percutaneous endoscopic gastrostomy.

In 81 (79.4%) of 102 patients, a feeding tube was inserted prophylactically at the end of the operation. In 45 (44.1%) patients, the feeding tube was removed after a mean of 12.6 days (SD, 15.6, 1–95). In the remaining 36 (35.3%) patients, the feeding tube was replaced by a PEG tube. Another three (2.9%) patients received a PEG tube before TLM-SGL and eight (7.8%) during later course. In summary, a total of 47 (46.1%) patients had a PEG tube at any time point during treatment. At 24 months follow-up, six (8.0%) of 75 patients had a PEG tube *in situ*. The PEG tube was removed at a mean of 284.2 days (SD, 224.0, 19–728). Out of 102 patients, a total of 41 (40.2%) had been tracheotomized. Among them, 35 (34.3%) were elective cases, while six (5.9%) were unplanned due to surgical complications. The prevalence of tracheostomies decreased continuously during the follow-up period: from 37 (36.3%) of 102 patients at 0.25 months follow-up to only four (5.3%) of 75 patients at 24 months follow-up. The time to the closure of the tracheostoma was a mean of 269.5 days (SD, 255.8, 3–762). The prevalence of PEG tubes and tracheostomas during follow-up is shown in **Figure 4**.

The highest prevalence of tracheostomies was noted at 0.25-month follow-up, with 16 (29.6%) of 54 nonirradiated vs. 21 (43.8%) of 48 (later) irradiated patients. The differences did not reach statistical significance at any time point during follow-up (all *p*-values > 0.05). The highest prevalence of PEG tubes was registered at 2 months follow-up, with 16 (32.7%) of 49 nonirradiated vs. 27 (56.3%) of 48 irradiated patients. Significantly higher prevalences were found in irradiated patients at 1 month (*p* = 0.025), 2 months (*p* = 0.013), and 6 months (*p* = 0.041) follow-up. At 24-month follow-up, the difference was no longer significant (*p* = 0.200).

### Swallowing- and voice-related QoL

**Figure 5:**
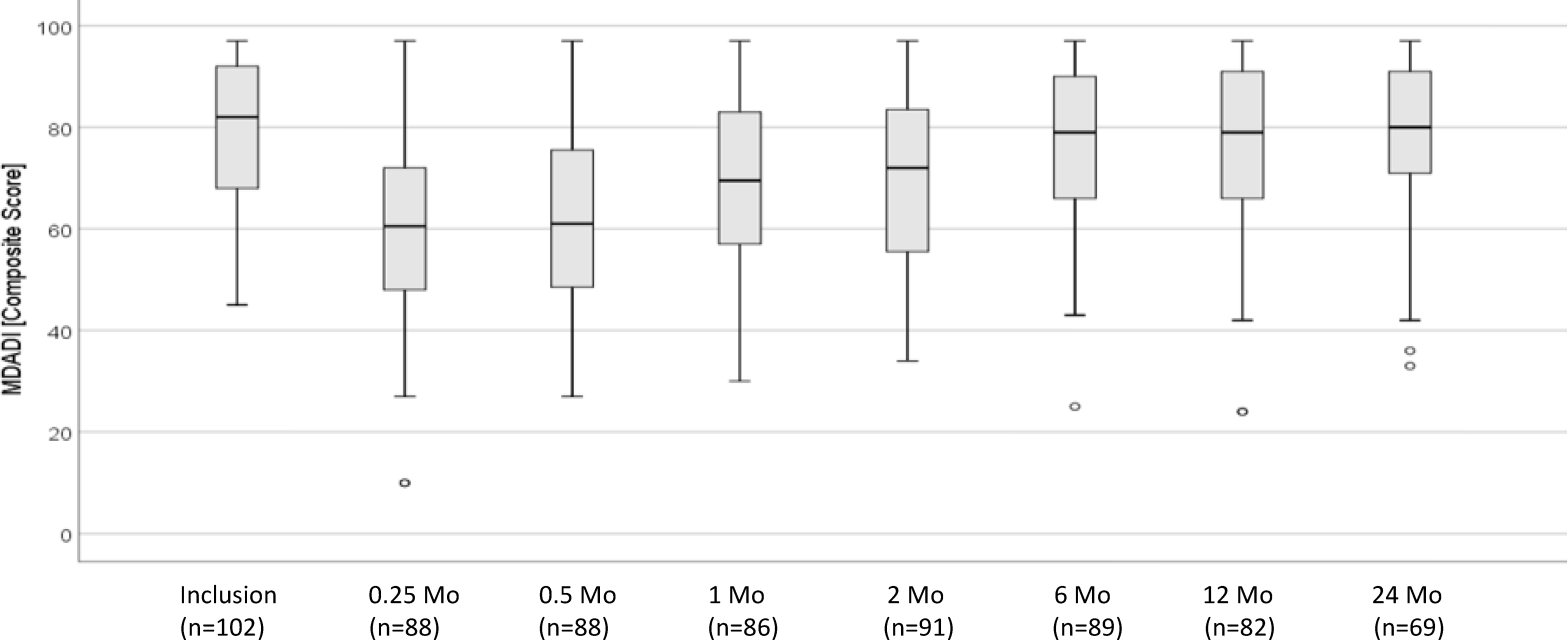
Longitudinal analysis of MDADI composite score (*n* = 102 patients). MDADI, MD Anderson Dysphagia Inventory.

**Figure 6:**
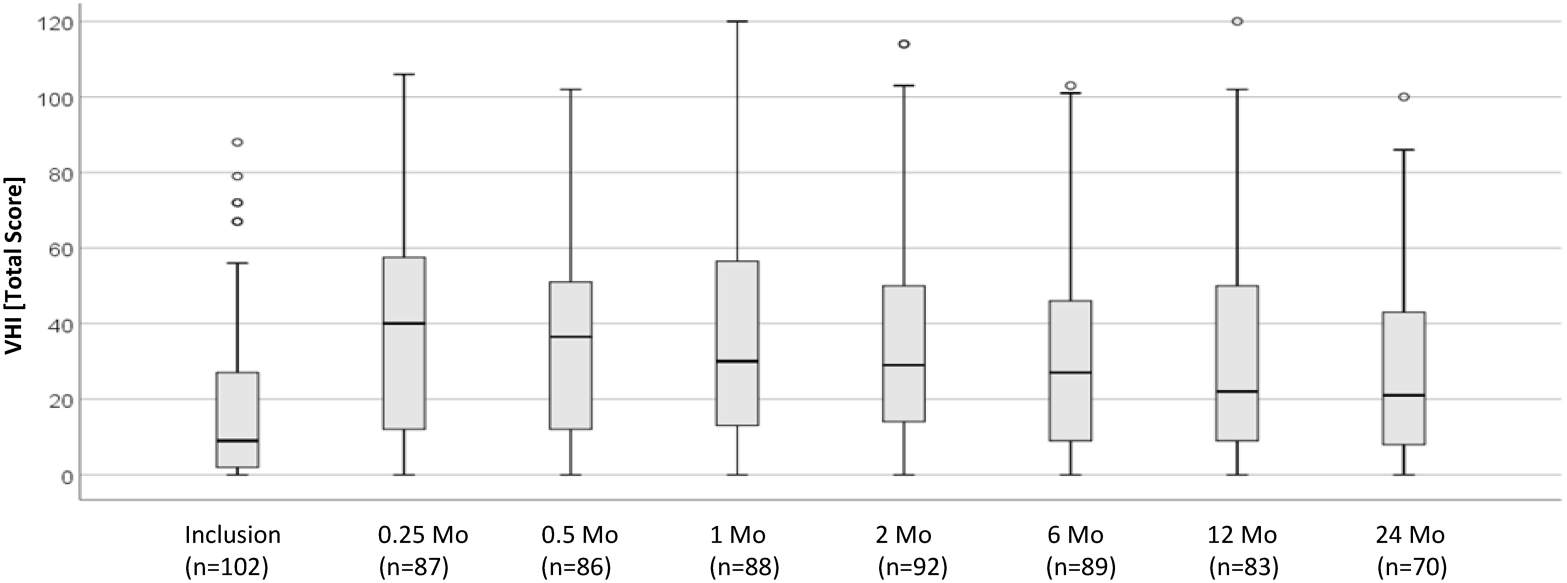
Longitudinal analysis of VHI total score (*n* = 102 patients). VHI, Voice Handicap Index.

The total return rate of MDADI and VHI questionnaires was 92.5% MDADI and 92.8% VHI questionnaires. All questionnaires were evaluable. At baseline, the MDADI composite score of 102 patients surveyed was a mean of 78.65 (SD, 15.01), and the VHI total score was a mean of 17.71 (SD, 21.04). During and immediately after treatment, at 0.25- to 2-month follow-up, the MDADI score dropped by a mean of 21.5 points, and after 6 months, the baseline values were reached again (**Figure 5**). At 0.25 to 2 months, the VHI score increased by up to mean of 17.99 points. After 12 months, an increase of 11.5 points compared with the mean baseline value was noted (**Figure 6**). The emotional, functional, and physical subscales of both instruments showed a comparable course (data not shown).

To examine the influence of adjuvant treatment (no irradiation vs. R(C)T), of the presence of a tracheostoma (present vs. absent), of a PEG tube (present vs. absent), and of the pT category (pT2 vs. pT3 tumor) on swallowing- and voice-related QoL, the mean MDADI composite scores and the mean VHI total scores, respectively, were compared for all follow-up visits. The comparison showed that already at the inclusion visit, the patients who had an indication for adjuvant R(C)T and/or were irradiated in later course had significantly lower MDADI scores (mean difference, 5.99 points; *p* = 0.046). Irradiated patients had significantly lower scores at 2-month (mean difference, 9.97; *p* = 0.004) and 6-month (10.38; *p* = 0.001) follow-up. At 12 months (7.28; *p* = 0.051) and 24 months (5.69; *p* = 0.113), differences were no longer significant. The MDADI scores of patients who had been tracheotomized were significantly lower at each postoperative follow-up visit (at 0.25 month, mean difference, 6.04; *p* = 0.084; at 0.5 month, 11.93; *p* = 0.002; at 1 month, 9.1; *p* = 0.029; at 2 months, 6.7; *p* = 0.021; at 6 months, 9.0; *p* = 0.021; at 12 months, 12.24; *p* = 0.005; at 24 months, 14.96; *p* = 0.023). The same applied to patients who had a PEG tube during treatment. Beginning at 0.5 month postoperatively, MDADI mean scores were significantly lower (at 0.5 month, 10.39; *p* = 0.014; at 1 month, 12.47; *p* < 0.001; at 2 months, 14.94; *p* < 0.001; at 6 months, 13.11; *p* < 0.001; at 12 months, 12.73; *p* = 0.008; at 24 months, 14.34; *p* = 0.016). The mean MDADI scores of patients with pT2 and pT3 tumors showed no significant differences at any time point during follow-up (all *p*-values > 0.05).

The VHI total scores of nonirradiated and irradiated patients were not significantly different at any time point (all *p*-values > 0.05). Patients with a tracheostoma had significantly higher mean VHI scores (at 0.5 month, 15.24; *p* = 0.017; at 1 month, 15.18; *p* = 0.027; at 6 months, 11.90; *p* = 0.043; at 12 months, 16.02; *p* = 0.023). Patients with a PEG tube had significantly higher VHI scores at 2 and 6 months (at 2 months, 16.32; *p* = 0.007; at 6 months, 13.38; *p* = 0.012). The comparison of the VHI total score in the groups of patients with pT2 and pT3 tumors showed no significant differences at any time point (all *p*-values > 0.05).

### Patterns of relapse and survival

Patients were followed with a median follow-up of 24.1 months. Local tumor recurrence was observed in 9.8% (10/102) of patients, among them one (1.0%) with concurrent disease in the neck. 4.9% (5/102) of patients with early recurrences (rT1) were successfully salvaged, four (3.9%) with further partial laryngeal resection, and one (1.0%) with definitive radiotherapy. At the end of the follow-up, all were free of disease and had a functioning larynx. Another 4.9% (5/102) of patients were diagnosed with rT3 or rT4 recurrences. In three (2.9%) patients, total laryngectomy was performed, and in two (2.0%), salvage treatment is unknown. One of the laryngectomized is alive and free of disease and two are lost for follow-up. The 2-year local control rate was 88%, and the 2-year rate of laryngectomy-free survival was 92%. Recurrence in the neck nodes, without concurrent disease at the primary site, occurred in 3.9% (4/102) of patients. Patients were treated with ND +/− RT. Two were successfully salvaged, one died of uncontrolled neck disease, and one died after salvage therapy from unknown cause. In 4.9% (5/102) of patients, a second primary tumor (SPT) developed. SPTs were located in the head and neck region in two (2.0%) patients: one patient with lateralized supraglottic carcinoma developed SPT in the contralateral piriform sinus and one in the soft palate. Both were successfully salvaged, were tumor-free at the end of follow-up, and had a functioning larynx. Two (2.0%) patients developed SPT in the lung and one (1.0%) in the colon. Overall, seven (6.9%) patients died: one (1.0%) tumor-related from neck recurrence, four (3.9%) tumor-unrelated, and in two (2.0%) patients, the cause of death is unknown. The 2-year rates for overall and for disease-free survival were 93% and 82%, respectively. Survival rates and corresponding 95% confidence intervals are shown in **Supplementary Table S5** in the **Supplementary Appendix**.

### Influence of treatment center on outcomes

According to the trial protocol (13), the oncological and functional outcome parameters should be stratified according to treatment centers. Stratification by participating centers was not possible due to the small number of recruited patients. Two centers were defined: the group of university hospitals (*n* = 16; 56.9% patients) and the group of nonuniversity hospitals (*n* = 10; 43.1% patients). No significant differences among treatment centers could be found for patient characteristics, except for alcohol consumption (*p* = 0.019) and previous cardiac diseases (*p* = 0.033), to the disadvantage of the university hospitals, for complications of surgical and adjuvant treatment, and for all oncological outcome parameters investigated. With regard to functional outcome parameters, the comparison of the dichotomized PAS revealed no significant differences between treatment centers at any time point and for all bolus consistencies tested (all *p*-values > 0.05). The comparison of the prevalence of a tracheostoma shows, however, that patients treated at nonuniversity hospitals were tracheotomized significantly more often together with the TLM-SGL than patients treated at university hospitals (at 0.25-month follow-up, 50.0% vs. 25.9%; *p* = 0.014). At 12-month follow-up (22.9% vs. 9.8%; *p* = 0.128) and at 24-month follow-up (12.9% vs. 2.3%; *p* = 0.153), the difference was no longer statistically significant. With regard to the insertion of PEG tubes, a significant difference between nonuniversity and university hospitals was seen at 0.25-month follow-up (22.7% vs. 6.9%; *p* = 0.039). No significant differences were noted in the later course (all *p*-values > 0.05).

## Discussion

### Accuracy of staging

The ability to predict the pathologic T classification is important for the success of partial laryngeal surgery. In particular, underestimation of the true tumor extent can be critical. The accuracy of the staging of supraglottic carcinomas was previously reported as 75%–85% (20–22). In our trial, cT staging was inaccurate in 25.5% of patients, resulting in upstaging in 10.8% of cases. The inaccuracies were within the expected range and did not have any negative consequences in retrospect. The comparison of cN and pN staging showed that 24.0% of patients with cN0 neck had occult metastases. The rate of occult nodal metastases in supraglottic cancer of all stages varies across recent studies from 14% to 23% (23–25). The up-front surgical approach allowed accurate pathologic staging. It was therefore possible to avoid radiotherapy in approximately 40% of patients. In the remaining patients, adjuvant RT, or RCT, was performed, depending on pathologic risk factors.

### Surgical quality and quality of adjuvant treatment

All TLM-SGL procedures were carried out as planned. Macroscopically incomplete (R2) transoral resection did not occur. No TLM-SGL had to be converted to an open procedure or terminated prematurely. Histopathological clear margins (R0 resection) were documented in 92.2% of cases, which corresponds to the numbers reported by high-volume centers (3, 4). However, the prevalence of clear margins differs considerably across institutional TLM-SGL series, varying from 42% to 92% (3, 4, 26, 27). Another parameter for the quality of a surgical procedure is the complication rate. In our trial, complications following TLM-SGL occurred in 7.9% of patients. Endolaryngeal hemorrhage was the most prevalent with 6.9%. All complications were managed successfully. The complication rate observed is within the reported range of 2%–14% postoperative bleedings (3, 6, 7, 9, 11). In deviation from the study protocol (13), the indicated adjuvant radiotherapy was not performed in 4.9% of patients and another 4.9% refused. The radiation doses to the larynx and to the neck corresponded to the doses specified in the trial protocol, with acceptable deviations. With regard to concurrent chemotherapy, protocol adherence was lower. In 25% of cases, institutional protocols were chosen. As far as side effects from adjuvant treatment are concerned, persisting dysphagia due to oesophageal strictures was observed in 4.2% of irradiated patients. In a recent analysis of the SEER-Medicare database, comprising 16,194 head and neck cancer patients, the prevalence of pharyngoesophageal stricture was 10.2% for all head and neck sites, following surgery alone, surgery with adjuvant RT, and R(C)T (28). In summary, it can be stated that the study protocol was adhered to with regard to the indication of TLM in 100%, ND in 96.1%, and adjuvant R(C)T in 89.7% of cases.

### Swallowing function

The examination of swallowing with FEES has proven to be feasible in this multicenter trial. However, it can be seen that the examination is complex and time-consuming, which is reflected in the number of examinations not done (mean, 8.3%) or not evaluable (mean, 9.5%). The compliance of both investigators and patients with patient-reported outcome measures was excellent, as visible in the very high return and evaluability rates (> 90%) of the MDADI and VHI questionnaires.

To the best of our knowledge, this is the first trial performing a longitudinal assessment of swallowing after TLM-SGL in a multicenter setting. Postoperative swallowing exercises were not prescribed because various techniques exist, but it is currently unclear which interventions may be associated with improved outcomes. The literature shows significant data heterogeneity and a paucity of objective measures for comparing functional outcomes of SGL. We assessed swallowing by standardized FEES using the PAS to describe the degree of dysfunction. The PAS is applicable after TLM-SGL since the anatomic landmarks needed for rating are endoscopically identifiable. When evaluating the results, it must be taken into account that the PAS is a descriptive ordinal scale and each score represents a unique categorical variable. Therefore, the scale is not suitable for arithmetic operations (29, 30). Previous research on the methodology showed that videofluoroscopy and FEES were equally effective in discriminating between penetration and aspiration (31). To exclude interobserver variability, all FEES were assessed centrally by one experienced reviewer (S.M.).

The evaluations showed that aspiration (PAS ≥ 6) was present in one-third of patients postoperatively, as to be expected. We could demonstrate that function improved steadily over time. Twelve-month follow-up was completed by 84.3% of patients. When tested with saliva, liquid, or pulp, 98.2%, 95.5%, and 98.8% of patients were free of aspiration. From the postoperative clinical observation of patients after TLM-SGL, it is known that increasing bolus viscosity reduces the risk of aspiration. The clinical observation was proven by this multicenter trial and confirmed previous clinical studies (32, 33). At long-term follow-up at 6 and 12 months, no lasting effect of adjuvant R(C)T, the type of supraglottic resection, pT category, presence of a tracheostoma, or presence of a PEG tube on swallowing function was detectable.

There are only a few studies that have investigated swallowing after TLM-SGL, most of them with small numbers of patients. Roh et al. (5) examined 21 patients after TLM-SGL for T1–3 supraglottic cancer preoperatively and up to 12 months postoperatively with videofluoroscopy. It was found that patients recovered from aspiration within 3–6 months. Piazza et al. (11) examined 36 patients after TLM-SGL for T1–3 supraglottic cancer with videofluoroscopy. Findings were rated using the Donzelli scale (34). Subglottic aspiration (corresponding to PAS ≥ 6) was detected in 7% of patients following limited and in 43% of patients following extended removal of supraglottic structures. Peretti et al. (35) compared 14 patients following TLM-SGL with 14 patients matched for the T category who were treated with open SGL. Endoscopic examination of swallowing 2 years after surgery demonstrated subglottic aspiration in 8% of patients following TLM-SGL and in 36% following open SGL. We could show that the swallowing function following TLM-SGL achieved in a multicenter setting was favorable and by no means inferior to the results published by specialized institutions.

### Patient-reported swallowing

It is generally assumed that clinically measurable swallowing impairment should be associated with poor QoL. However, the congruence between clinician-rated and patient-perceived function is still not well understood, and previous research shows conflicting results. It was found that the PAS and the MDADI scores correlate poorly (36–40). However, when the PAS was stratified between no aspiration/penetration (PAS < 6) and aspiration (PAS ≥ 6), a difference in MDADI scores was observed (41). Others found aspiration diagnosed with videofluoroscopy (42) or FEES (43) to be associated with lower scores on a variety of QoL scales. In a cohort of open SGL patients, the MDADI was found to show significant diagnostic accuracy only in the detection of moderate to severe pharyngeal residue and severe dysphagia diagnosed with FEES and scored with the PAS (44). Significant associations between FEES scored with the modified barium swallow impairment profile and MDADI scores were found in another study only in the first postoperative weeks (45). The results of our trial indicate that with the MDADI, severe swallowing dysfunction can be reliably recognized.

Our trial is the first prospective study about swallowing- and voice-related QoL of patients treated for supraglottic carcinomas. The longitudinal assessment showed, during and immediately after treatment, a clinically significant drop by a mean of 21.5 points (MDADI) or an increase by a mean of 18.0 points (VHI). The analysis of the MDADI and VHI scores at 6- and 12-month follow-up demonstrates that patients have to accept no losses in swallowing-related QoL and minor losses in voice-related QoL in the long term. From other studies on functional outcomes after treatment of head and neck cancer, it is known that patient-reported difficulties with swallowing increase within the first 3 months after completion of therapy and decrease from 6 months onwards, although the pretreatment values have not always been reached (46–48).

Of interest is the fact that in our trial, patients irradiated postoperatively had significantly worse swallowing- and voice-related QoL during treatment, but they recovered from that, and in the long term, no differences between irradiated and nonirradiated patients were observed. As to be expected, the swallowing-related QoL of patients who had been tracheotomized or had been supplied with a PEG tube was significantly lower at any time point. In our trial, tracheostomy and PEG tube had a longer-lasting impact on QoL than adjuvant R(C)T. The pT category had no influence on swallowing- or voice-related QoL. The reason might be that the extent of resection is similar in pT2 and pT3 tumors.

### Tracheostomy and gastrostomy

With open SGL, all patients have to undergo tracheostomy along with laryngeal surgery. Tracheostomy is required at a much lower frequency in TLM-SGL. In our trial, 40.2% of patients required a temporary, mostly elective (85.4%) tracheostomy. The rate of elective tracheostomy in this trial was unexpectedly high. The reported incidence of temporary tracheostomy from all causes in different TLM-SGL series varies between 4% and 29% (3, 5, 6, 8, 9, 11, 49, 50) and between 4% and 14% in high-volume centers (3, 8, 9). Known reasons influencing the decision for elective tracheostomy are bleeding and mucosal swelling during surgery, simultaneous bilateral neck dissection, the anticipated need for adjuvant R(C)T, and institutional preference. In our trial, a significant influence of adjuvant R(C)T on the prevalence of tracheostomies could not be demonstrated. It is noteworthy, however, that patients treated at nonuniversity hospitals underwent elective tracheostomy twice as often as patients treated at university hospitals. As there were no differences between the two treatment centers in terms of patient characteristics, treatment, and functional and oncologic outcomes, it can be supposed that structural reasons might have been relevant in the decision-making process. The prevalence of definitive tracheostoma was 5.3%, which corresponds to the reported prevalence of 3%–8% in the retrospective TLM-SGL series (8, 11, 49–52). In this trial, no patient required a secondary total laryngectomy for persistent aspiration. The rate of secondary laryngectomy ranges from 0% to 6% in the retrospective TLM-SGL series (3, 6, 8, 9, 11, 50).

Our data clearly show that gastrostomy tubes were widely used for supportive therapy during adjuvant R(C)T. A significant influence of adjuvant R(C)T on the prevalence of PEG tubes was found at 1-, 2-, and 6-month follow-up. The prevalence declined to 8.0% at 24 months. The rate of chronic gastrostomy dependence in the TLM-SGL series ranges from 1.3% to 2.2% (3, 8, 9). However, this outcome parameter is not reported for all retrospective cohorts. In a systematic review of 10 retrospective cohort studies on functional outcomes of partial laryngeal resection (open SGL, TLM-SGL, and TORS-SGL) vs. radiotherapy for early supraglottic cancer, the pooled event rate for “intractable aspiration” and “permanent gastrostomy tube dependence” was 2.6% (95% CI, 1.0%–6.8%) and 5.3% (95% CI, 2.6%–10.5%), respectively, in the surgically treated cohort. This parameter was not reported for the radiotherapy group in any cohort study (12).

### Preliminary oncologic outcomes

In the trial cohort, 62.7% of patients had pT2 and 26.5% pT3 tumors. The pathologic stage distribution was 40.2% of patients with stages I and II and 59.8% with stages III and IVa disease. According to the trial protocol (13), the postoperative follow-up time was set to 24 months. Interpretation of the oncologic results is therefore limited. In previous reports, the oncologic results of single-institution TLM-SGL cohorts as well as the results of R(C)T have been reviewed repeatedly (1, 3, 9, 10, 53, 54). All preliminary oncologic results achieved in our multicenter trial, are in accordance with the figures reported in the literature. Kaplan-Meier estimates for local control, laryngectomy-free survival, overall survival and disease-free survival are shown in **Supplementary Table S5** in the **Supplementary Appendix**. The 2-year local control rate was 88%, corresponding to 83% for pT1 and pT2 and 72% for pT3 supraglottic tumors, as reported previously (3). The 5-year local control rates for T1 and T2 tumors range from 85% to 97% and 74% to 82% for T3 tumors (2–4, 8, 9, 52). The 2-year laryngectomy-free survival rate in this trial was 92%. In most studies, the 5-year larynx preservation rate was reported and quantified as 79%–97% for pT2 and 74%–89% for pT3 tumors (2, 3, 9, 52). In our trial, the 2-year overall and disease-free survival rates were 93% and 82%, respectively. The 2-year overall and disease-free survival rates were previously reported as 90% and 75% for stages I and II and 75% and 71% for stages III and IVa disease (3). The analysis shows that local control, laryngectomy-free survival, and survival rates in our multicenter trial did not differ from mono-institutional TLM-SGL cohorts.

### Future perspectives

In recent years, TORS for SGL has received increasing attention, and several retrospective cohort studies (55–57) have been published. In a systematic review (58) comprising 14 studies, treatment results of 422 patients were described. Patients treated with TORS-SGL had mainly cT1 and cT2 tumors; only 13.9% had cT3 tumors. Despite remarkable heterogeneity between studies regarding the inclusion/exclusion criteria, treatment of the neck, and adjuvant treatment modalities, the evidence currently available suggests that TLM and TORS are comparable in terms of morbidity (use of feeding and PEG tubes, the prevalence of temporary tracheostomy) and oncological outcomes (2-year local control and overall survival rates). Data on swallowing function and swallowing- and voice-related quality of life that would be comparable to those obtained in our trial are not available. To investigate which treatment option is most effective in terms of functional outcomes, a prospective randomized trial comparing the three treatment modalities TLM-SGL, TORS-SGL, and R(C)T would be desirable.

## Limitations

Our trial has some limitations. It was not possible to recruit the targeted number of 200 patients, resulting in limited options for subgroup analysis. FEES has been quite complex and time-consuming, leading to a significant number of examinations that are not fully evaluable or are missing.

## Conclusion

In the authors’ opinion, this multicenter trial demonstrates that with TLM-SGL + (S)ND +/− R(C)T for the treatment of supraglottic carcinomas, good functional results can be obtained. It was shown that more than 95% of patients achieved aspiration-free swallowing. Preliminary oncologic results are consistent with previous single institutional data. Morbidity in terms of temporary elective tracheostomy and PEG tube placement was higher than expected. However, permanent tracheostoma and chronic gastrostomy use were within the previously reported range. Examination of swallowing- and voice-related QoL demonstrates that patients have to accept only minor losses in QoL in the long term. This is the largest report in the literature on the functional outcomes of TLM-SGL.

## Supplementary information on the dynamics of swallowing rehabilitation

### Method

To examine the dynamics of swallowing rehabilitation over the course of the trial at the individual level, scores were referenced to the baseline PAS at the pre-operative examination. For evaluation, three categories were formed: Increase in PAS by 2 or more PAS-points (deteriorated function); unchanged or change by 1 PAS-point up or down (unchanged function); decrease in PAS by 2 or more PAS-points (improved function).

### Results

After the first postoperative week, deteriorated function is seen in 59.0%-64.3% of patients depending on the bolus consistency tested. After 2 months, unchanged function is seen for saliva and liquid in 63.0% and 70.5% patients, respectively. For the bolus consistency pulp, a stabilization of the swallowing function can already be seen one month post-operatively. With pulp, 68.7% of patients show unchanged function compared to the score at baseline. This reflects the clinical observation that semi-solid boluses are swallowed earlier and better than thin liquids. At 12 months follow-up unchanged or even improved swallowing function was observed in 82.5%-88.1% of patients and at 24 months follow-up in 84.0-92.5% of patients depending on the bolus consistency tested. Results are shown in **Supplementary Figures S1A–C** in the **Supplementary Appendix**. To examine the influence of adjuvant treatment (no irradiation vs. R(C)T), presence of a tracheostoma (present vs. absent), and presence of a PEG tube (present vs. absent) on the dynamics of swallowing rehabilitation, the mean dichotomized PAS for saliva, liquid and pulp were compared. Comparisons at all follow-up visits showed no statistically significant differences at any time point and for all bolus consistencies (all p values >0.05).

### Conclusion

The assessment of the dynamics of swallowing rehabilitation over the course of the study on an individual level showed that one week postoperatively impaired function compared to function at baseline was observed in 61.8% of patients when tested with liquid and in 59.0% of patients when tested with pulp. On the other hand, this means that 40% of patients had no relevant impairment of swallowing function in the immediate postoperative phase. At 12 months follow-up, impaired swallowing function was still observed in 16.1% of patients when tested with liquid and in 11.9% when tested with pulp. In other words, about 85% of tested patients did not suffer from long-term dysphagia due to the cancer treatment. The difference in the analysis of swallowing function at the individual level, compared to the results of dichotomized PAS can be explained by the identification of events that must be rated as penetration and not as aspiration (increase in 2 or more PAS-points, but PAS <6).

## Data Availability

The raw data supporting the conclusions of this article will be made available by the authors upon request.

## Supplementary material

Supplementary Table 1 Local principal investigators and participating hospitals.

Supplementary Table 2 Baseline characteristics of patients (detailed version, n=102 patients).

Supplementary Table 3 Treatment modality (n=102 patients).

Supplementary Table 4 Results of histopathologic examination and pathologic staging (detailed version).

Supplementary Table 5 Kaplan-Meier estimates (n=102 patients).

Supplementary Figure 1 Longitudinal analysis of the change of dichotomized PAS referenced to PAS at baseline in %. **(A)** Tested. Saliva, PAS, penetration-aspiration scale. **(B)** Tested: Liquid, PAS, penetration-aspiration scale. **(C)** Tested: Pulp, PAS, penetration-aspiration scale.
