# Supplementary material for: Transoral laser microsurgery for supraglottic carcinomas: results of a prospective multicenter trial (SUPRATOL)
Source: Front Oncol. 2024 Sep 20;14:1440024. doi: 10.3389/fonc.2024.1440024 (PMC11449847; doi:10.3389/fonc.2024.1440024)
Supplement: Supplementary Table 1 — Local principal investigators and participating hospitals. [file DataSheet1.pdf]

Supplementary Appendix

Table S1 Local principal investigators and participating hospitals

PI	Participating Hospital
P. Ambrosch, A. Fazel	Dept. of ORL-HNS, University Hospital Schleswig-Holstein, Campus Kiel and Christian-Albrechts-University Kiel, Kiel
A. Dietz	Clinic of ORL, University of Leipzig, Leipzig
U. Schroeder	Dept. of ORL-HNS, University Hospital Schleswig-Holstein, Campus Luebeck and Luebeck University, Luebeck
A. Lammert	Dept. of ORL-HNS, Mannheim Medical Faculty of Ruprecht-Karl-University Heidelberg, Mannheim
J. Kuenzel	Dept. of ORL-HNS, University Medical Centre of Johannes Gutenberg University, Mainz
M.C. Jäckel	Dept. of ORL, Helios-Kliniken, Schwerin
D. Boeger	Dept. of ORL, SRH Zentralklinikum, Suhl
C. Scherl	Dept. of ORL-HNS, Friedrich Alexander University, Erlangen-Nuremberg, Erlangen
T. Deitmer	Dept. of ORL-HNS, Klinikum Dortmund, Dortmund
K. Breitenstein	Dept. of ORL, Helios-Klinikum, Erfurt
K.-W. Delank	Dept. of ORL-HNS, Klinikum der Stadt, Ludwigshafen
H. Hilber, S. Vester	Dept. of Otorhinolaryngology, Head and Neck Surgery, University Hospital, Regensburg
S. Knipping	Dept. of ORL-HNS, Städtisches Klinikum, Dessau
U. Harreus	Dept. of ORL, Evangelisches Krankenhaus, Düsseldorf
M. Scheich	Dept. of ORL, Plastic, Aesthetic and Reconstructive Head and Neck Surg., Julius-Maximilians-University Hospital, Wuerzburg
S. Bartel, S.K. Plontke	Dept. of ORL-HNS, Martin Luther University Halle-Wittenberg, Halle (Saale)
S. Koscielny	Dept. of ORL-HNS, University Hospital, Jena
J.A. Veit, J. Greve	Dept. of ORL-HNS, Ulm University Hospital, Ulm
V. Schilling	Dept. of ORL-HNS, Vivantes Klinikum Neukölln, Berlin-Neukölln
M. Linxweiler	Dept. of ORL-HNS, Saarland University, Homburg
S. Weiß	Dept. of ORL, Klinikum, Kassel
G. Psychogios	Dept. of ORL-HNS, Klinikum Augsburg Süd, Augsburg
C. Arens	Dept. of ORL-HNS, Otto-von-Guericke University, Magdeburg
C. Wittekindt	Dept. of ORL-HNS, University of Giessen, Giessen
J. Oeken	Dept. of ORL, Hospital Chemnitz, Chemnitz
M. Grosheva	Dept. of ORL-HNS, University of Cologne, Cologne

ORL-HNS, Otorhinolaryngology-Head and Neck Surgery,
PI, principal investigator

Table S2 **Baseline characteristics of patients (detailed version, n=102 patients)**

**Characteristic**	
**Age** [yrs], Mean (SD), Min, Max	61.9 (8.21), 42.0, 82.0
**Sex**	
Male, n (%)	70 (68.6)
Female, n (%)	32 (31.4)
**Smoking history**	
Never smoker, n (%)	5 (4.9)
Active smoker, n (%)	71 (69.6)
Former smoker, n (%)	26 (25.5)
**Pack years***, Mean (SD), Min, Max	37.9 (17.07), 5.0, 98.0
Missing, n (%)	9 (8.8)
**Alcohol history**	
Abstinent, n (%)	32 (31.4)
Active alcohol consumption, n (%)	69 (67.6)
Unknown, n (%)	1 (1.0)
**ECOG performance status, n (%)**	
0	91 (89.2)
1	9 (8.8)
2	1 (1.0)
3	1 (1.0)
**Comorbidity**	
COPD, n (%)	23 (27.5)
Cardiac disease, n (%)	33 (32.4)
Cerebrovascular disease, n (%)	17 (16.7)
Metabolic disorder, n (%)	20 (19.6)
Missing, n (%)	1 (1.0)
Renal disease, n (%)	5 (4.9)
Missing, n (%)	1 (1.0)
**cT category**, n (%)	
T1	3 (2.9)
T2	76 (74.5)
T3	23 (22.6)
**cN category,** n (%)	
N0	54 (52.9)
N1	15 (14.7)
N2a	4 (3.9)
N2b	18 (17.7)
N2c	9 (8.8)
N3	2 (2.0)
**Clinical UICC stage,** n (%)	
I	1 (1.0)
II	45 (44.1)
III	23 (22.6)
IVa	33 (32.3)
**Preoperative vocal fold mobility,** n (%)	
Normal	83 (81.4)
Unilaterally impaired	13 (12.7)
Bilaterally impaired	1 (1.0)
Unilateral fixed	3 (2.9)
Not assessable	2 (2.0)

*Information on pack years refers to active smokers (n=71)

Table S3 Treatment modality (n=102 patients)

**Modality**	
**Transoral Laser Microsurgery**, n (%)	
Medial type resection	58 (56.9)
Lateral type resection	44 (43.1)
**Neck dissection**, n (%)	
Unilateral	27 (26.5)
Bilateral	71 (69.6)
No neck dissection	4 (3.9)
**Type of Neck dissection**, n (%)	
Modified radical	8 (4.7)
Selective	161 (95.3)
**Adjuvant treatment,** n (%)	
None	54 (53.0)
Radiotherapy	24 (23.5)
Radiochemotherapy	24 (23.5)

Table S4 **Results of histopathologic examination and pathologic staging (detailed version)**

**Parameter**	
**Typing**, n (%)	
Keratinizing SCC	48 (47.1)
Non-keratinizing SCC	44 (43.1)
SCC	10 (9.8)
**Grading**, n (%)	
G1	5 (4.9)
G2	50 (49.0)
G3	45 (44.1)
Missing	2 (2.0)
**Re-resection**, n (%)	
Done	15 (14.7)
Not done	85 (83.3)
Missing	2 (2.0)
**Resection status**, n (%)	
R0	94 (92.2)
R1	3 (2.9)
R2	0
Rx	5 (4.9)
**Minimal distance of tumor to resection line [mm]**, n (%)	
0	3 (2.9)
1-5 („close resection margins“)	43 (42.2)
≥5	36 (35.3)
Missing	20 (19.6)
**pT category**, n (%)	
pT1	11 (10.8)
pT2	64 (62.7)
pT3	27 (26.5)
**pN category**, n (%)	
N0	4 (3.9)
pN0	53 (52.0)
pN1	12 (11.8)
pN2a	3 (2.9)
pN2b	20 (19.6)
pN2c	10 (9.8)
**Pathologic UICC stage,** n (%)	
I	7 (6.9)
II	34 (33.3)
III	28 (27.5)
IVa	33 (32.3)

Table S5 Kaplan-Meier estimates (n=102 patients)

Parameter	2-year rate (%)	95% confidence interval
Local control	88	77.1; 98.92
Laryngectomy-free survival	92	86.7; 97.3
Overall survival	93	84.9; 101.1
Disease-free survival	82	68.9; 95.1

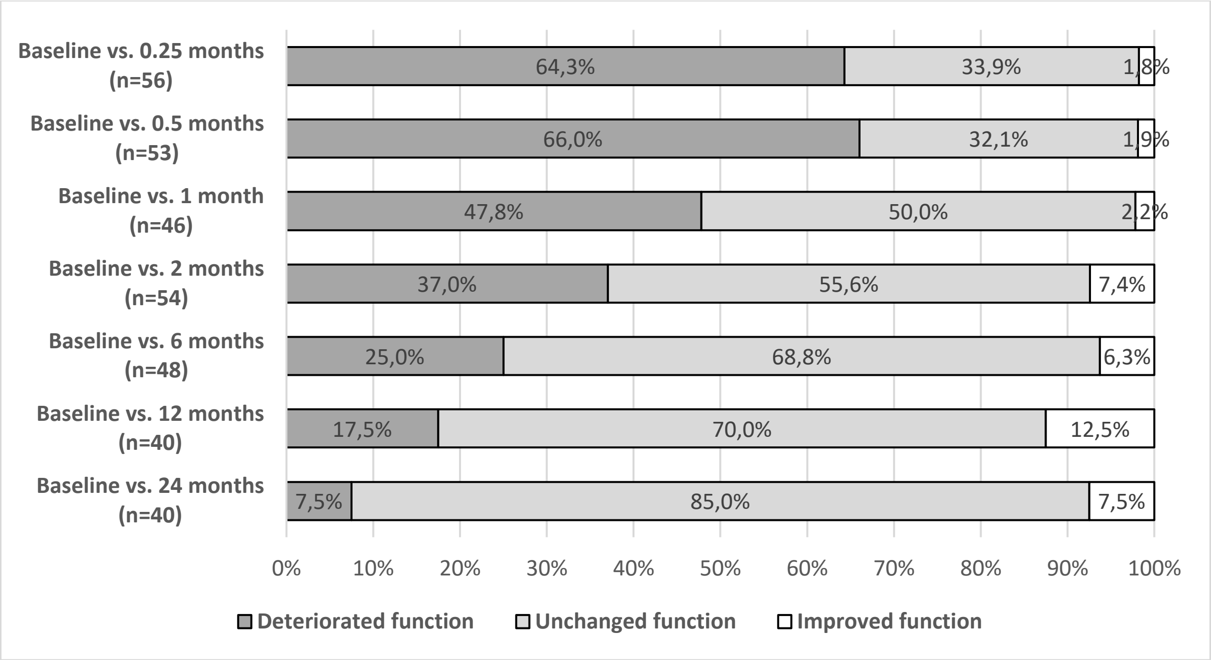

Figure S1 (A) Longitudinal analysis of the change of dichotomized PAS referenced to PAS at baseline in %.
Tested: Saliva, PAS, penetration-aspiration scale

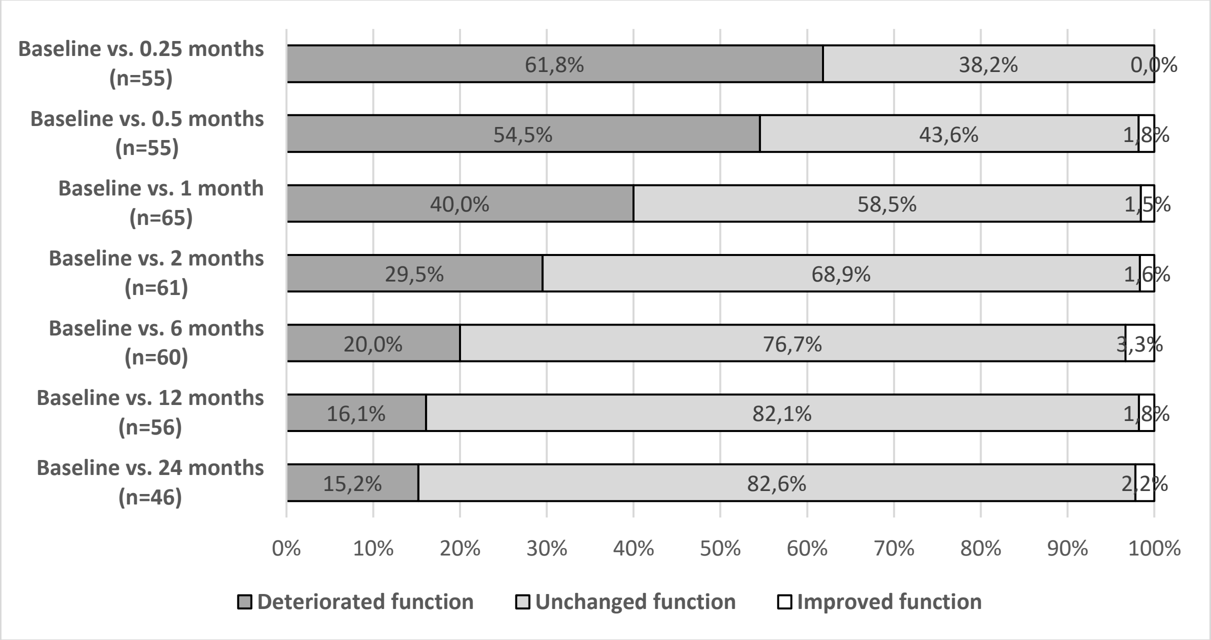

Figure S1 (B) Longitudinal analysis of the change of dichotomized PAS referenced to PAS at baseline in %.
Tested: Liquid, PAS, penetration-aspiration scale

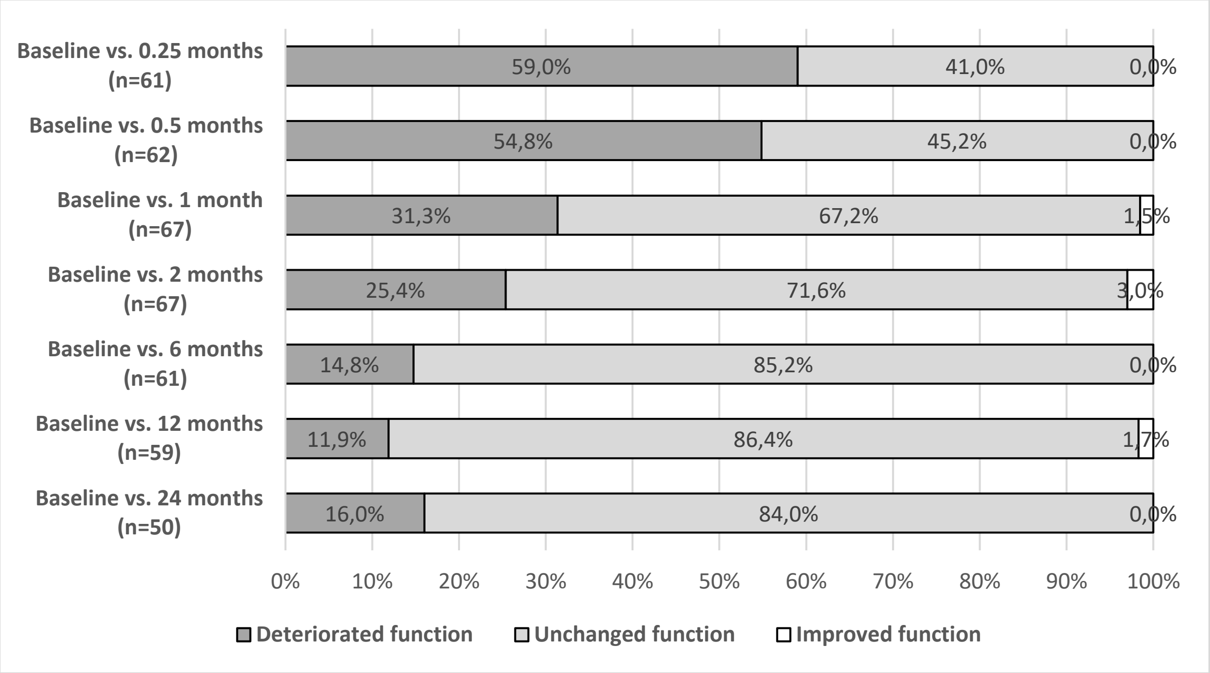

Figure S1 (C) Longitudinal analysis of the change of dichotomized PAS referenced to PAS at baseline in %.
Tested: Pulp, PAS, penetration-aspiration scale
